# The Swiss Preschoolers’ health study (SPLASHY): objectives and design of a prospective multi-site cohort study assessing psychological and physiological health in young children

**DOI:** 10.1186/s12887-016-0617-7

**Published:** 2016-07-08

**Authors:** Nadine Messerli-Bürgy, Tanja H. Kakebeeke, Amar Arhab, Kerstin Stülb, Annina E. Zysset, Claudia S. Leeger-Aschmann, Einat A. Schmutz, Fady Fares, Andrea H. Meyer, Simone Munsch, Susi Kriemler, Oskar G. Jenni, Jardena J. Puder

**Affiliations:** Endocrinology, Diabetes & Metabolism Service, Centre Hospitalier Universitaire Vaudois (CHUV), Lausanne, Switzerland; Department of Clinical Psychology and Psychotherapy, University of Fribourg, Fribourg, Switzerland; Child Development Center, University Children’s Hospital Zurich, Zurich, Switzerland; Children’s Research Center, University Children’s Hospital Zurich, Zurich, Switzerland; Epidemiology, Biostatistics and Prevention Institute, University of Zurich, Zurich, Switzerland; Department of Psychology, University of Basel, Basel, Switzerland; Division of Pediatric Endocrinology, Diabetology and Obesity, Centre Hospitalier Universitaire Vaudois (CHUV), Lausanne, Switzerland

**Keywords:** Child, Preschool, Stress, Physical activity, Development, Longitudinal, Cognition, Motor skill, Psychology, Health

## Abstract

**Background:**

Children’s psychological and physiological health can be summarized as the child’s thinking, feeling, behaving, eating, growing, and moving. Children’s psychological and physiological health conditions are influenced by today’s life challenges: Thus, stress exposure and lack of physical activity represent important health challenges in older children. However, corresponding evidence for young children is scarce. The aim of *Swiss Preschoolers’ Health Study (SPLASHY)* is to examine the role of stress and physical activity on children’s psychological and physiological health, particularly on cognitive functioning, psychological well-being, adiposity and motor skills in children at an early stage of childhood. We will also assess the role of child and environmental characteristics and aim to define sensitive time points.

**Methods/design:**

In a total of 84 child care centers, children at preschool age (2–6 years) are recruited and are assessed immediately and one year later. Assessments include direct measurements of the children in the child care centers and at home as well as assessments of children’s behavior and environmental factors through informants (parents and child care educators).

**Discussion:**

*SPLASHY* is one of the first studies in early childhood aiming to investigate the influence of stress and physical activity on children’s psychological and physiological health in a community-based longitudinal design.

**Trial registration:**

Current Controlled Trials ISRCTN41045021 (date of registration: 21.03.14)

## Background

For the first time in history, children have a shorter lifespan than their parents due to obesity and non-communicable lifestyle-related chronic disease [1]. Improving children’s overall health represents a major goal for researchers, practitioners and policy makers. General health in young children includes high levels of cognitive functioning and social skills, psychological well-being, a healthy body weight, and well developed motor skills. These domains can be summarized as the child’s thinking, feeling, behaving, eating, growing and moving in an optimal way, even under challenging conditions.

Stress and lack of physical activity (PA) represent two relevant health challenges in today’s modern environment that may interfere with children’s health [2, 3]. Exposure to environmental stressors (ranging from major severe life events to daily stress) is omnipresent. On the other side, lack of physical activity has become the 4th leading cause of death worldwide. To better elucidate the impact of these two factors, alone and in combination, on children’s health, prospective studies starting in young children are needed. Consequently, SPLASHY covers health outcomes of various domains such as cognitive functioning, psychological wellbeing, body weight and motor skills at important developmental transition time points during childhood. Additionally, parenting style and family’s exposure to critical live events and daily stress are considered.

### Importance of the chosen health outcomes

*Cognitive functioning* is fundamental for a healthy development of the child assuring the mental processing of information (i.e., the construction of human thoughts or mental processes). It includes attention, memory, inhibition, and more complex functions such as producing and understanding language, solving problems and making decisions [4]. Cognitive functioning is a predictor for school and academic performance determining the career [5].

*Psychological wellbeing:* Psychological problems and mental disorders are common during childhood [6, 7]. They impact substantially on quality of life as well as on family and school functioning of the child [6] and induce an increased risk for later suffering from mental disorders [6]. Central to the concept of psychological wellbeing are the regulation of mood and eating behavior and the occurrence of behavior problems as they affect a broad range of developmental tasks and the daily functioning of the child [6].

*Adiposity or absence of healthy body weight:* Around 20 % of European and US children are overweight or obese and prevalence rates are even over 30 % in some countries [8–10]. Childhood obesity and overweight carry a considerable health burden, namely cardiovascular, orthopedic, reproductive, gastrointestinal, neurological and psychological problems and a high BMI in childhood is associated with a 40–60 % increase in risk of all- cause mortality in adulthood [11, 12].

*Motor skills* are an essential component for developmental processes of children and influence cognitive or emotional aspects of children’s health (see e.g., [13, 14]). Normally developed motor skills are important for children’s daily activities and their participation in social engagements. In contrast, impaired motor skills and motor coordination problems may lead to poorer self-efficacy and lower life satisfaction in school-age children and adolescents (see for a comprehensive review [15]).

### Impact of stress on children’s psychological and physiological health

**Fig. 1:**
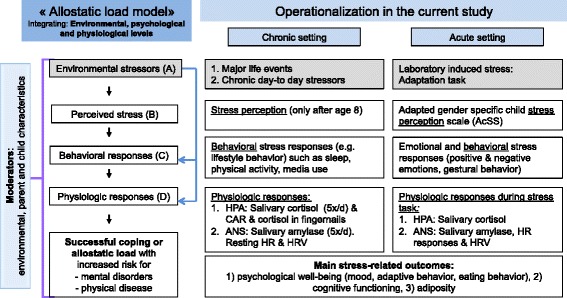
Stress concept and operationalization in the current study

*Stress* is a universal condition of human existence. The concept of allostasis (see Fig. 1, left column) describes the active process by which humans adapt to environmental stressors in order to maintain homeostasis and promote survival [16–18]. Stress occurs when environmental stressors exceed and dysregulate the adaptive capacity (allostasis) of an individual resulting in psychological and physiological changes. These changes may lead to disturbances in mental and physical health (allostatic load) [17, 18] (see Fig. 1).

Environmental stressors (A) are part of children’s life and include major life events, but also chronic day-to-day stressors. In children, chronic day-to-day stressors can comprehend socially disadvantaged family situations, chronically poor parental health, low parental involvement, exaggerated parental worries concerning children’s health and high parental stress [19–21].

The ability to adjust to environmental stressors is influenced by the way one perceives this challenge. Stress perception (B) leads to behavioral responses (C) such as “fight or flight” responses, lifestyle behaviors or adaptive coping behavior and can be evaluated in the acute and chronic setting. Stress perception is also linked to physiological stress responses (D), the most common responses involving the hypothalamic-pituitary-adrenal (HPA) axis and the autonomic nervous system (ANS) that respond both to chronic and acute stress situations. These two stress systems interact with each other and with other mediators (neurotransmitters, cytokines etc.) in a complex non-linear network and impact on different processes such as CNS function, metabolism and cardiovascular function [16]. Serum or salivary cortisol concentrations are the most frequently used biomarkers of the activity of the HPA axis. The ANS can be divided into the parasympathetic and the sympathetic nervous system (SNS) and both influence heart rate (HR) and heart rate variability (HRV) as indicators of cardiac response to stressors. Recent data show that salivary alpha-amylase concentration can be a reliable and useful marker of the activity of the SNS which stimulates acinar cells of the salivary glands via beta-adrenergic receptors [22, 23]. Diurnal cortisol release is typically characterized by high levels on waking, peaking at approximately 30 min (called the cortisol awakening response, CAR), and a subsequent decline over the remainder of the day both in adults and children [24, 25]. On the other side, salivary alpha-amylase concentrations are low at waking and increase over the day [23], but data in children are lacking.

The exposure to repetitive environmental stressors (chronic stressors) can for example alter the diurnal patterns of the biomarkers of the main physiological stress systems, i.e. of both salivary cortisol and alpha-amylase [23, 26]. As another example, a high resting HR and/or reduced HRV present an ANS imbalance with decreased parasympathetic activity. Although still explorative, cortisol levels in the fingernails may be indicative of chronic environmental stress exposure, as cortisol is able to diffuse passively from the capillaries into the human nail matrix and become keratinized [27]. In general, several types of physiological stress responses can result in maladaptive allostatic load such as 1) frequent stressors with lack of adaptation possibly leading to 2) failure to shut-down the persistent elevation of stress hormones or 3) inadequate response with failure to respond to challenge [18]. For example, exposure to persistently dysregulated secretion of stress hormones can subsequently contribute to cognitive impairment, mental disorders and obesity [16, 17, 28–30] (see also below). Similarly, any imbalance in ANS activity with decreased parasympathetic activity is related to metabolic complications, poor cognitive function and emotional dysregulation. Thus, a lower HRV can predict greater psychological problems in children, while a higher HRV serves as a buffer against detrimental effects of chronic stressors [31, 32]. In the acute setting, acute stress reactivity to a specific stressor can be expressed as changes in salivary cortisol or alpha-amylase concentrations (albeit with different response times) or in increases in the HR and decreases in the HRV in response to a standardized acute stressor [33–36]*.* The level of acute stress reactivity shows the individual sensitivity or the differential susceptibility to an environmental stressor and can indicate a risk for the development of allostatic load. For example, acute stress reactivity has been associated with childhood adiposity [37]. Thus, chronic exposure to environmental stressors in early childhood may have long-term health consequences and acute stress reactivity may have an additional impact. Several factors such as parent (e.g., familial atmosphere and positive parenting styles influencing developmental processes) and child characteristics (e.g., genetic predisposition, temperament, physical health) and early stress experiences moderate the response to stressors and can thus help to explain the outcome variability in stress responses. Thus, multiple factors determine if a stressor leads to successful adaptation, coping and resilience or to allostatic load.

*Stress and cognitive functioning:* Environmental stressors and the subsequent activation of both main physiological stress systems, that is the HPA axis and the ANS, can have impairing or enhancing effects on memory, attention and executive functioning. These differential effects depend on several factors such as age, type and time course of the stressor and the moderating role of the environmental context and support [38–40]. Chronic stress exposure may have an impact on neurocognitive functioning, especially memory and selective attention [40]. Data suggest that early and continuous stress exposure might particularly affect the frontal cortex and possibly also the hippocampus, but studies in human are controversial [40]. Acute stress (such as witnessing a threatening situation) reduces children’s performance on vocabulary and reading assessments for 2–3 weeks [41]. In experimental settings, acute stress exposure seems to impair memory retrieval [38]. However, there are only few data in children and there is a lack of knowledge about more long-term cognitive effects of heightened stress reactivity or of chronically high or low cortisol or SNS activity.

*Stress and psychological wellbeing:* Repeated or enduring exposure to stressors seems to be crucial for mental health [42]. Chronic (family) stressors, in contrast to episodic stress seem to be linked to a gene-environment interaction with youth possessing special high-risk alleles being prone to develop depressive symptoms [42]. Recent data show that individual differences in stress reactivity such as the organism’s capacity to respond to acute and prolonged stressors may be associated with the development of anxiety and mood disorders [43]. However, data assessing stress response in different systems such as physiological, behavioral and emotional domains is scarce. Another well-known but unsolved issue is the question about the impact of the experience of stress and stress response patterns at different ages through early childhood. Hence, it is of high relevance to investigate how the experience of stress and the child’s individual stress reactivity relate to the (later) development of psychological well-being and to define possible moderators.

*Stress and adiposity:* The exposure to environmental stressors and the psychological and physiologic stress responses have been related to childhood obesity, but there are very few longitudinal data and data in young children are needed [20, 21, 30, 37, 44]. Urinary free cortisol, urinary cortisol metabolites and morning plasma cortisol as markers of the HPA axis have been associated with BMI, total or central body fat in some cross-sectional studies [28, 29]. However, as far as we know, there are no longitudinal data relating plasma or salivary cortisol levels to BMI in children. A chronic stimulation of the HPA axis and subsequent increase cortisol exposure in concert with a concurrent cortisol-stimulated elevation in insulin concentrations can increase body fat accrual and could lead to (central) obesity, insulin resistance and the metabolic syndrome. Studies in children are needed to further investigate if cortisol is also directly related to increased body fat or if stress might act through behavioral changes such as emotional “comfort” eating, impulsive behaviors, selection of specific foods, lack of sleep and a decrease in physical activity [30, 45, 46] There is further a need for studies relating the activity of the ANS to obesity in children which is a largely understudied field.

### Impact of physical activity (PA) on children’s psychological and physiological health

From a developmental perspective, body motion is a fundamental condition for adequate motor and cognitive development as a precondition for children’s global well-being. Since the spreading burden of chronic disease, PA has gained a pivotal role in the prevention of psychological [47, 48] and physical [49] diseases and is believed to improve cognitive functioning [50]. This is especially relevant as recent studies indicate that young children are insufficiently active with 3–5-year old children spending around 80 % of their time in activities classified as sedentary or at most as light PA [51].

*PA and cognitive functioning:* Epidemiological research supports experimental findings in animals that (especially aerobic) exercise can enhance human brain structure, prevent structural tissue loss and improve cognitive performance and academic achievements [52]. Diamond [53] has summarized the current evidence that the prefrontal cortex and the cerebellum are co-activated during movements and cognitive tasks suggesting that they are equally important for both motor and cognitive functions. Yet, few investigations are currently available which have reported inconsistent findings in terms of the magnitude and nature of the motor-cognition association during childhood (see e.g., [13, 54, 55]). A single study exists in preschool children performed by an interdisciplinary collaboration of the current co-applicants, in which higher baseline aerobic fitness and motor skills were related to a better spatial working memory and/or attention at baseline, and to some extent also to their future improvements [56]. Ackerman [57] and Voelcker-Rehage [58] were suggesting in his model of motor learning that there is a positive relationship between motor skills and cognitive functioning in the preschool period which is thought to decrease with age as motor skill levels increase.

*PA and psychological well-being:* PA interventions in the general population of children have a positive impact on self-esteem and beneficial effects on anxiety and depression scores [47]. There is evidence that the level of PA in childhood might protect against depressive symptoms in adults, although additional prospective longitudinal studies are needed [59]. We have shown that the effect of PA on quality of life is especially pronounced in obese children [60, 61]. As most studies are cross-sectional and there is an inconsistency between measures, the relationships between PA and mental health is still an underinvestigated area of research and even less is known on the processes linking PA and psychological well-being. A current review encourages additional research investigating the moderating role of family atmosphere and child characteristics [48] on the association between PA and psychological well-being.

*PA and adiposity:* Most studies using objectively measured PA found inverse relationships between PA and BMI or body fat, but few data exist in preschoolers and longitudinal studies show controversial results [62, 63]. Yet, lifestyle interventions including PA performed in preschool or school age children had a beneficial effect on body fat [64–66].

*PA and motor skills:* While we know quite well how motor skills develop from early childhood into school age (see e.g., [67]), detailed knowledge about the driving forces for this development is scarce. Intuitively, PA and repetitive motor training would be the main forces. This idea builds on the hypothesis that practice through PA training may lead to an increase in synaptic strengths that is observed when motor skill training is performed [68, 69]. Thus, the PA level of young children may tell us whether they have sufficient amount of practice (i.e., PA) or not. In cross-sectional and longitudinal studies, PA has been positively related to different motor skills in school [70] and preschool children [63]. Similarly, PA intervention studies in preschoolers [65, 71, 72] have demonstrated an improvement in motor skills. However, the amount of prospective studies is scarce, particularly in children below the age of 5 years.

*PA and stress:* The beneficial effect of PA on physiological and mental disorders [52] is at least in part explained by a reduction in stress responses, resulting in reduced stress hormones levels [73], higher self-esteem [47], improved cognitive factors [74] and improved physiological health with better fitness, less adiposity, and reduced cardiovascular stress reactivity [75, 76]. Regular PA also leads to a higher parasympathetic activity, which beside other influences is thought to be one health-protecting mechanism [77, 78]. Yet, men who were highly responsive to exercise stress (concerning HPA-axis) were also highly responsive to psychological stress, suggesting that there may be a genetic trait determining responsiveness to stressors [79]. To our best knowledge, data on acute stress response in terms of HPA axis and HRV changes (i.e. changes in physiological parameters in response to challenging conditions) in children are lacking.

### Moderating role of psychological child and environmental characteristics

Psychological child and environmental characteristics are related to environmental stressors, stress responses and PA, but also to psychological and physiological health outcomes. The process by which children (psychological child characteristics) regulate and their caregivers (psychological parent characteristics) co-regulate stress responses has not yet been given sufficient attention in literature (see e.g. [6]). It is therefore important to take these moderators into account when studying the relationship of stress and PA with children’s health. Other factors such as physiological health (PA, adiposity, health problems) and developmental experience can, where appropriate, also act as moderators.

### Psychological child characteristics

Psychological child characteristics such as temperament, self- or emotion regulation are related to the child’s ability to cope with acute or chronic stressors [80]. For example, temperament determines changes in cortisol concentrations in children visiting child care [81]. Child characteristics are also related to psychological well-being, adiposity, motor development and cognitive functioning. Self-regulation capacity represents a general determinant of later competences to control one’s behavior, thoughts, motor abilities and emotions [82]. We have shown that correlates of self-regulation capacities are associated with parental behavior (especially with mothers’), with different styles of eating behavior [83], with PA [84] and have also been related to adiposity [85].

### Environmental characteristics (parents, child care, large environment)

*Family setting:* Familial socioeconomic status (SES, including migrant status, educational level and income), parenting style, family atmosphere, parental role modeling such as their health attitude, behaviour and support for a healthy lifestyle can all affect children’s PA levels and physiological and mental health [86–88]. Salivary cortisol has been reported to be higher in children with lower SES and may be mediated by a greater exposure to stressful life events [89]. We have shown that parental SES factors were related to obesity in young children [90–92]. In addition, psychological parent characteristics such as parenting style [93] and family atmosphere [94] have a major impact on children’s behavioral responses in stressful situations.

*Child care/ (pre)school setting:* Differences in child care quality and health promotion in the child care/school setting can impact on children’s health. Child care centers/schools are essential determinants for PA as well as for developing motor skills, physical fitness and a healthy body weight, as we had confirmed in our intervention studies [64, 65]. The quality of child care can impact on the salivary cortisol levels [81] as well as on cognitive functioning and behavior [88, 95].

*Large sociocultural/neighborhood environment*: Differences between neighborhoods and within Europe highlight the impact of the sociocultural environment for lifestyle factors such as PA or obesity in children. Similar to the well-known European North–south-decline in PA and the corresponding increase in obesity [96], differences in adult PA levels between the different sociocultural regions (north-eastern German vs south-western French speaking part of Switzerland) have been reported within Switzerland [97]. In a previous study we have confirmed this observation in preschool children [90].

### Potential sensitive time points

Evidence is accumulating that early life conditions affect long-term health outcomes [98] and this early impact on health may limit the effectiveness of later interventions. The earlier in life stressful life events occur (the period up to age 5 seems to be especially sensitive), the more important their (long term) health impact may be, as a permanent biological embedding of developmental processes into regulatory physiological processes may take place (“programming effect”) [98, 99].

Up to now, there is a lack of longitudinal studies that include both potentially challenging and health promoting predictors and their effect on both psychological and physiological health outcomes. Our study fills this gap and offers the opportunity to gain insights in potential sensitive periods where certain fundamental health indicators might be particularly receptive (e.g., for PA promotion) or vulnerable (e.g., to stress exposure). In conclusion, it remains open how relevant determinants of today’s life, stress and PA, in combination or by interaction with different child’s and environmental characteristics, influence psychological and physiological health, i.e. how they think, feel, behave, eat, grow and move at a given and at future time-points. In conclusion, the expected study results will contribute to a global assessment of children’s health for research, practice and policies and thus as a basis for targeted prevention and early intervention.

## Method/design

### Aims

The overall aim of *Swiss Preschoolers’ Health Study (SPLASHY)* is to investigate how stress (as a paradigm for a potentially health challenging predictor) and PA (as a paradigm for a potentially health promoting predictor) influence children’s psychological and physiological health by focusing on four essential health outcomes, i.e. cognitive functioning, psychological well-being, adiposity and motor skills. The main hypotheses for the respective research questions are the following:

1. Chronic exposure to environmental stressors (major life events, chronic day-to-day stressors), chronic physiological stress responses (dysregulations of the hypothalamic-pituitary-adrenal (HPA) axis, dysregulation of the autonomic nervous system (ANS), and the dysregulation of acute laboratory induced stress reactivity to a standardized stressor) are associated with/predict increased current and prospective adiposity, reduced cognitive functioning, and reduced psychological health (i.e., emotional problems, behavioral problems and dysfunctional eating behavior scores).
2. High levels of total physical activity and more time spent in moderate-to-vigorous physical activity correlate with/predict current and prospective lower levels of adiposity, better cognitive functioning and motor skill performance and increased psychological health.
3. Stress exposure and physical activity have an impact on psychological and physiological stress responses.
4. Aspects of child’s development and health (cognitive and emotional development, self-regulation skills, adiposity, motor skills) are related to each other.
5. The strength of all these associations vary with age.
6. Environmental characteristics such as *sociocultural environment* (German vs French part of Switzerland), *child care center characteristics* (quality, health promoting activities), *parental characteristics* (family atmosphere, parenting style, parenting stress and family lifestyle, parental BMI, socioeconomic status), *psychological child characteristics* (temperament, emotion and self regulation, pre- and postnatal conditions*),* and *physiological child characteristics* (lifestyle behavior, adiposity) impact on the above mentioned outcomes and have a moderating role in the relationship between stress, PA and psychological and physiological health of preschool children. It is further assumed that the strength of association varies with age and that children’s characteristics (lifestyle, temperament, emotional well being and self regulation) and environmental characteristics (family atmosphere, parental style, socioeconomic status, child care center characteristics and the sociocultural environment such as German-French speaking parts of Switzerland) may moderate the relationship of stress and physical activity on psychological and physiological health outcomes.

### Design

SPLASHY is a prospective cohort study including children during early childhood within two sociocultural areas of Switzerland (German and French speaking part). The project uses a multi-site approach including four research groups recruiting child care centers within five cantons of Switzerland (Aargau, Bern, Fribourg, Vaud, Zurich) which together made up 50 % of the Swiss population in 2013.

### Study sample

Sample size calculations were based on the data simulation software MLPowSim 2 in combination with R, version 2.13.1, with 5000 permutations per simulation. We thereby selected an effect size *r* = 0.18 which was the minimum expected effect size in any sub-project in order to have a sufficiently high sample size to test hypotheses in any sub-project. Further conditions were: statistical power 1–β = 0.8, α = 0.05, two-sided test.

Thus, the minimal sample size to have sufficient power at the third assessment wave was calculated to be 240 children. For reasons of feasibility, we planned to recruit 96 child care centers (24 for each of the four research groups). Based on a previous study [100] we presumed that around 12 children per child care center or 1150 children in total would be present and could be invited on a given afternoon of testing. We assumed a participation rate of 40 % or 500 children at T1 with 5.2 children per child care center. In addition, we assumed a worst case scenario in which the analysis was based on data from the third assessment wave (T3, not part of the current study design), where an additional 48 % of the data was expected to be missing. This estimated dropout rate of 48 % was based on a dropout rate of 20 % between T1 and T2 and another 40 % dropout between T2 and T3 resulting in a final sample size at T3 of 240 children. The higher dropout of 40 % between T2 and T3 is based on the situation that merely all children will have left the child care center by this time and will go to kindergarten elsewhere.

Child care centers were recruited in five cantons Aargau (AG), Berne (BE), Fribourg (FR), Vaud (VD), Zurich (ZH) which together contained 50 % of the Swiss population in 2012 [101]. The selection procedure was stratified according to one stratum with four levels: urban community and rural community with high SES (above-average) and low SES (below-average) each based on the prevalence of child care centers in the respective communities. For FR and BE, all child care centers were invited to participate due to a low number of existing child care centers in these cantons.

Urban communities were defined as the biggest cities of each canton as well as cities >100’000 inhabitants. This included Zurich and Winterthur (ZH), Bern (BE), Fribourg (FR), Aarau (AG), Lausanne (VD). All other communities were defined as rural communities.

Socioeconomic status was defined by the maternal education level of the community based on the report of the 2010 SNF ‘NFP 60 “familienergänzende Kinderbetreuung und Gleichstellung” (REF [102]). Thus, child care centers for which the community’s proportion of mothers with a university degree was higher or lower than the median were accordingly defined to have high or low SES, respectively.

For the larger urban communities, a different method for SES stratification was chosen as the community SES would not represent it appropriately. The list of child care centers was divided into high and low SES using the definition of SES according to the Swiss neighbourhood index of socioeconomic status [103]. Thereby, deciles of the index were constructed and high SES was defined as the deciles 6–10 and low SES as the deciles 0–5.

**Fig. 2:**
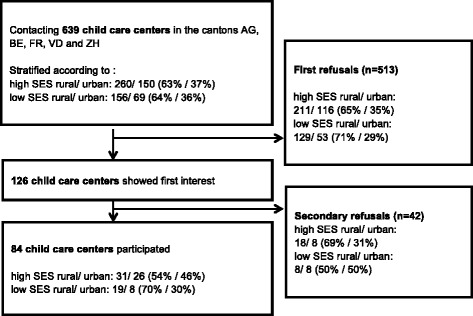
Recruitment and participants flow

A total number of 639 child care centers in the French and German part of Switzerland were contacted in the period between January 2013 and October 2014 by four different research groups and were informed on study participation (see Fig. 2). Of these, 126 child care centers agreed to participate and to inform the parents. Reasons for refusals were lack of time (26 %), too few (less than 4) children of the selected age group present in child care on a given afternoon (21 %), no interest (21 %) and organizational changes (13 %). Further, 42 centers had to be excluded after the preparation of testing dates due to too few (less than 2) participating children (78 %) or for other reasons (12 %). The final sample consisted of 84 child care centers that participated, which is 12 % less than the originally planned number of 96 child care centers.

In addition, instead of the expected number of 12 children per child care center, a mean of 7.6 children (2–6 years old) were present at a given assessment afternoon and were invited to participate. The participation rate for these was, however, much higher than expected (73.9 %, *n* = 476 at T1). In summary, expected and real recruitment numbers differed by 24 children in the total sample. Therefore, for T2, new arriving children of the same child care centers are currently still recruited in order to obtain the desired number of children at T2 and T3. Note that the number of 240 children at T3 leads to slightly increased power when coming from 84 rather than 96 child care centers because of the reduced design effect. However, this beneficial effect is expected to be very small.

### Inclusion and exclusion criteria

To attain the largest external validity possible, we aim at including as many children as possible thereby keeping exclusion criteria at a minimum. We will inquire about any medication intake and existing acute and chronic health problems (e.g., allergies, asthma) and passive smoke exposure and record this information. We will exclude children below the age of 2 and above the age of 6 and for certain testing or analyses on a case to case basis according to the question studied if there may be a possible interference between their health or treatment and the testing (e.g., intake of inhaled steroids for the cortisol measures) or if the child is unable to perform the test (e.g., motor handicap).

### Assessment

All direct measurements at baseline and one year later are conducted in the child care centers on three subsequent afternoons between 1.30 p.m. and 6 p.m. of the same weekday (adiposity & motor skills; self-regulation and cognitive functioning; acute stress reactivity). In addition, measures of chronic stress (heart rate variability overnight, salivary cortisol and alpha amylase over one weekday and one weekend day, cortisol in fingernails) and of physical activity over one week are assessed, questionnaires are completed by parents and child care educators and parents conduct an interview on family atmosphere by using the Five Minutes Speech Sample [104]. An overview of all measurements is given in Table 1. Measurements are divided into testings of children during the three assessment afternoons in child care and at home (direct testing) and assessments with parents and child care educators as informants (indirect testing).

**Table 1:** Outcomes and measures. Children’s direct and indirect (parent and child care educator assessment)

Measures	Tool	informants
Indirect assessment by parents or child care educator		
Major life events, chronic day-today stressors, SES, neighborhood, lifestyle, pre-& perinatal conditions, birth weight, breastfeeding, early regulatory problems, general health, reported PA	General health questionnaire	parents
Parenting style	Alabama Parenting Questionnaire (APQ)	parents
Parental stress	Parental Stress Scale (PSS)	parents
Children’s eating behavior	Children’s Eating Behavior Questionnaire (CEBQ)	parents and child care educators
Children’s mood and behavior problems	Strengths and Difficulties Questionnaire (SDQ)	parents
Children’s temperament	Emotionality Activity Sociability Temperament Survey (EAS)	parents
Children’s emotion regulation	Index combining EAS scales, observed emotion regulation behavior, salivary cortisol, HRV during adaptation task	parents and child
Family atmosphere	Parental Expressed Emotions by Five-Minute Speech Sample (FMSS)	parents
Social contacts (with peers)	Child care questionnaire	child care educators
Children’s direct assessment at home		
Physical activity	Accelerometers	child
Physiological stress responses in the chronic setting	Salivary amylase & cortisol, clipped fingernails, HR and HRV	child
Children’s direct assessment at the child care center		
Afternoon A:		
Adiposity	BMI, sum of 4 skinfolds, waist circumference	child
Motor skills	Zurich Neuromotor assessment (ZNA3–5)	child
Afternoon B:		
Cognitive functioning	Intelligence and Development Scales-Preschool (IDS-P) – 4 cognitive tests	child
Self regulation	Statue test (NEPSY)	child
Afternoon C:		
Stress response/acute stress reactivity	Adaptation task with stress perception, behavioral responses, salivary amylase & cortisol, HR & HRV, Picture Stress Test	child

Follow-up measurements one year later include the same assessments. Each center defined a team with one responsible and 2 to 3 assistants per afternoon for the testing sessions. Testings for the first period have all been done between February 2014 and November 2014 in parallel in different child care units for the baseline testing and during the follow up one year later further children within the same child care centers were recruited between February 2015 and November 2015. Before starting the testing period all assistants were trained in assessment techniques on three different days. Quality check of testings were performed every 3 week by analyzing videotaped testing sessions of each testing site by an expert team.

### Measures

Assessment of stress includes a chronic and an acute perspective and covers several parts of the allostatic load model such as environmental stress exposure, perceived stress, behavioral responses, physiological stress responses as well as the successful coping or allostatic load including an increased risk for mental disorders or/and physical diseases (see Fig. 1).

#### Chronic setting

The chronic setting involves environmental stressors and physiological stress responses. *Environmental stressors* (parental report as part of the general health questionnaire) include major life events and chronic day-to-day stressors. Major life events will be assessed by a total score of single items including death, serious disease, accident of a relative or close friend, divorce, exposure to violence/abuse (major negative life events based on the Coddington Life Events Scale CLES [105]. In a second step a subjective impact rating of each event (intensity score 1–3) as previously described will be done [106]. Chronic day-to-day stressors included parental unemployment [20], socially disadvantaged situations, parental worries concerning child’s health [107], daily hassles including problems and conflicts, separations of parents, low parental involvement by the Alabama Parenting Questionnaire [100], high parental stress (Parental Stress Scale PSS) [108], and poor parental health [19]. Physiological stress responses will be assessed through HR and HRV, salivary cortisol and alpha-amylase, cortisol in fingernails. Resting HR and HRV will be measured and analyzed based on standard procedures over the measurement period with a eMotion HRV (Mega Electronics, Kuopio, Finland) by fixing two electrodes on the chest of the child that are connected to a little box (35x35x15mm) that is worn and fixed over the upper chest [109]. A methods paper for the measurement of HR and HRV in preschoolers will be published separately. Measurement of basal salivary cortisol and alpha-amylase concentrations (during one weekday with child care and one weekend day; 5 times/day) include: upon awakening in the morning around 7.30–8.00 (within 10 min of awakening), 30 min after awakening (cortisol awakening response- CAR), before lunchtime (11.30–12.00), before snack (16.00) and at bedtime (20.00). Parents enter the time periods of assessment in a diary. Salivary cortisol and alpha-amylase samples are collected using Salivette (Sarstedt) collection devices, which are cotton rolls that children keep in their mouth for 1 min. The salivettes are collected by the trainees from the child care centers at the respective sites and are stored at −20 °C until badge analysis in the freezer of each center. Salivary cortisol is analyzed using a commercial chemiluminescence immunoassay (LIA) (IBL Hamburg, Germany) and alpha-amylase using an automatic analyser Cobas Mira (assay kits from Roche), as previously described [110].

The measure of cortisol in fingernails is still explorative. We therefore assess and validate in this project this innovative and perhaps simpler approach to cortisol assessment. Nail clippings are washed, dried for two hours at 45° Celsius and then cut into small pieces of 1–2 mm. After further purification, cortisol levels in nails are determined by enzyme immunoassay (EIA) [27].

### Acute setting

Acute stress reactivity is assessed according a laboratory stress induction paradigm using an age - appropriate adaptation task entailing a socially evaluative component, perceived uncontrollability and motivated performance according to Kryski et al. [33].

Standardized environmental stressor (adaptation task)*:* The original protocol of Kryski et al. [33] was adapted slightly in terms of icons (frogs and ladybirds instead of ball icons). Additionally an animated picture of a traffic stoplight on a computer screen was integrated. Feasibility and acceptance of the testing was established in a pilot study. The adaptation task is applicated in a separate room and children are asked to sit together with a non-familiar experimenter at a table in front of a large felt board with numerous ladybirds and butterflies affixed and colored stones (blue and red). At the beginning, the child chooses a prize from an assortment of small toys. The experimenter explains to the child that each of the icons have to be covered by a stone and the child is given some time to practice prior to starting the task. The child is then told that there is plenty of time to work when the light is green (2 min 20 s), but when the light turns yellow, time is running out and when the light turns red in combination with a loud buzzer (after another 40 s), time is up. The red light is accompanied by a loud buzzer sound. The child is told that the adaptation task is easy to be done and even small children are able to finish on time, but that the child must match all the animals on the board with the stones to receive their preferred prize. The time limitation is manipulated by the testing person to allow challenging situations which are comparable for all the children. Three subsequent identical trials are carried through in which children are unsuccessful and the chosen present is refused. After the third trial, the experimenter exclaims that the light was broken and that the child had not been given enough time. Therefore, the child’s matching skills are praised and the child receives his preferred toy.

Based on a pilot study, salivary protocol described by Kryski et al. [33] has been adapted. Cortisol and alpha amylase is assessed at 7 different time points in total and HR and HRV will be continuously measured before the beginning of the adaptation task and up to 75 min after the end of the task using eMotion HRV. In order not to interfere with the impact of the diurnal rhythm as well as PA or food intake, we perform testing in the early afternoon (starting between 1.30 and 3.30 p.m.), at least 60 min after waking up from day nap, at least 90 min after lunch and at least 60 min after a light snack eating, or drinking as done in the study of Kryski et al. in the same age group [33]. Children are not allowed to be physically active in between lunch and this testing.

The *child’s stress perception* is assessed using an age - adapted nonverbal test (Picture Stress Test) using the response format of the Harter scales [111]. This adapted child stress perception scale assesses feelings of distress using gender specific pictures (e.g., sadness, anxiety, tension, shame, guilt, anger/aggression). For each item, two pictures are presented simultaneously. One depicts the critical symptom, while in the other picture the symptom is not shown (neutral). The child is asked which rabbit he or she resembles and indicates the degree of his or her feeling on a 4-point-scale [112]. Videotaped *emotional and behavioral stress responses* including facial, verbal and physical display of children’s positive and negative emotions (PE and NE) are assessed and coded using visual analogue scales (VAS) ranging from 0 to 5 (0 = no PE/NE or low intensity/number of expressions of PE/NE or 5 = high intensity/number) according to the Kryski protocol [33]. Baseline PE and NE measures are assessed during the introduction of the task and immediately after stress induction when the child is told that it runs out of time. Changes in baseline PE and NE relative to stressful events will be calculated by subtracting summed instances and intensity of emotions expressed during the introduction from that expressed during the stressful period of the task thereby controlling for the duration of these two periods. PA levels will be rated using a 0 to 5 VAS with anchoring points (very low PA to high PA).

Physical activity is monitored with an accelerometer (MTI/CSA wGT3X+, Actigraph, Shalimar, FL, USA), which children continuously wear around the hip for five weekdays and two weekend days during each measurement period. ActiGraph wGT3X+ is a triaxial accelerometer that is used to measure the amount and frequency of accelerations of the body. The monitor is small and lightweight, measures 4.6 cm* 3.3 cm* 1.5 cm and weighs 19 g. The ActiGraph monitors are affixed above the iliac crest of the right side of the hip with an elastic belt. Data acquisition storage is set at 3-s epochs. The output includes activity counts (vertical x, horizontal y, and diagonal z axes), vector magnitude, which is equal to the square root of ((amplitude x)^2^ + (amplitude y)^2^ + (amplitude z)^2^), and number of steps taken. Times of 20 min and more of 0 readings will be removed and considered as non-wear time. Each sample will be summed over a 15-s or 60-s epoch according to validation studies for preschool children [113, 114] to determine the level of overall PA (in counts per minute, cpm), and time spent in different levels of PA as recently suggested [115, 116]. For an initial evaluation, individual child’s physical activity data will be accepted as valid if at least three weekdays (at least one day at the child care center) and one weekend day of measurements with a minimum of 10 h are recorded. Children that do not fulfill these quality criteria are remeasured. We also assess the time when the child gets up and goes to bed to assess time awake and control compliance. Each statistical model will be controlled for differences in wearing time. Further, a questionnaire is used to assess the extent and pattern of children’s physical activity including sports club and leisure time sports participation, as well as parental support for and attitude towards physical activity.

Three different measures are used to assess adiposity: Body mass index, body fat and waist circumference. To define body mass index, standing height and body weight are measured using an electronic scale (Seca, Basel, Switzerland; accuracy 0.05 g) and BMI percentiles are calculated for age and gender of each child. Overweight and obesity will be also pooled for some analyses due to the relatively low prevalence at this age, labeled together as “overweight” and defined according to WHO criteria [117], the International Obesity Task Force (IOTF) criteria [118, 119] and the national Swiss percentiles [120, 121]. Total body fat is assessed by skinfold measurements. The skinfold thickness is measured in triplicate to the nearest 0.1 mm with Harpenden calipers (HSK-BI, British Indicators, UK) calibrated to exert a pressure of 10 g/cm2 to the skin. A maximal difference of 1 mm between the 3 measurements will be allowed for data to be valid. Four sites (triceps, biceps, subscapular and suprailiac) are measured based on standard procedures and the sum of four skinfolds calculated [122]. Waist circumference (midway between the iliac crest and the lowest border of the rib cage) is measured in duplicate to the nearest 0.1 cm by a flexible tape. A maximal difference of 0.5 cm between the 2 measurements will be allowed for data to be valid. Waist circumference is an indicator for central body fat.

Cognitive functioning is assessed by using the IDS-P (Intelligence and Development Scales- Preschool) [123]. The advantage of this test battery is its development in a Swiss population and the possibility to use it in our longitudinal design not having to change test items for different ages with a version for 3- to 5- year-old and then for 5- to 10-year-old children allowing longitudinal comparisons. The full test battery is used to determine the state of development of cognitive functioning as well as general development in psycho-motor abilities, social-emotional competency, mathematics and language as well as performance motivation. For the younger age group, internal consistency (Cronbach α) ranged from 0.55–0.96, and was around 0.8, except for some single exceptions. For the older age group of 5- to 10-year-old children correlation between general intelligence scores measured by IDS and HAWIK-IV was 0.83 [124]. The test battery has a high construct validity for age trends, shows high intercorrelation of individual scales and factor loading, as well as criterion validity for the HAWIK-IV and differential validity for giftedness or children with attention-deficit-hyperactivity disorder. As cognitive testing in preschool is not evident, simple reliable measures that cover major executive functions and have been shown to be responsive to physical activity as described in the above cited studies were selected. These include four tests described in detail in the IDS Manual [123]: visual perception, selective attention, visual special working memory, figural reasoning. We will base our analysis on a normalized summary score that is calculated by summing the age and gender based IDS z-scores of the respective tests.

Psychological well-being: Parents are asked to respond to different questions regarding mood, temperament, behavioral problem, eating behavior and self-regulation capacities of their child. We assess parents’ ratings of child’s emotional and behavioral problems using the Strengths and Difficulties Questionnaires (SDQ [125]). Parents’ rating of the child’s eating behavior and temperament is assessed using the Child Eating Behavior Questionnaire (CEBQ [126]) and the Emotionality Activity Sociability Temperament Survey (EAS [127]) respectively. Additionally, the child care educators evaluate the eating behavior of the children according to the CEBQ [126]. Further, a correlate of self-regulation is assessed by the statue test of the Neuropsychological Assessment for Children, NEPSY [82], where the child is told to stand still like a statue, while the experimenter tries to attract attention of the child.

Emotion regulation as the ability to modify the valence, intensity, or time course of emotional reactions will be assessed as suggested by Adrian et al. [128] in a multi-method approach. This includes the parent’s ratings of emotionality and sociability of the EAS [127], physiological markers (delta baseline-to max salivary cortisol and HR) self-rated and observed emotion regulation behavior (Stress perception according to AcSS; PE, NE/gestures/emotion regulation categories) assessed during the laboratory stress induction paradigm: the child’s gestural behavior will be analyzed according to the NEUROGES-ELAN coding system [129] and gestures directed towards the body and self-touch gestures reflecting emotion regulation behavior will be coded. The NEUROGES coding inventory consists of objectively defined kinetic and functional movement categories, developed based on neuropsychological gesture research of recent decades. The ELAN annotation tool has been proved to be a reliable instrument for assessing gestural behavior. Further, trained raters will estimate emotion regulation categories such as cognitive change, emotional support, instrumental intervention, instrumental support, attentional deployment, response modulation and denial according to Gross & Thompson [130]. For this purpose, a coding system will be developed.

Motor tests will be performed by trained testers of the ZNA3–5 [67] and the ZNA5–18 [131] which has been developed to assess fine and gross motor functioning in young and older children. The ZNA5–18 is a well-standardized motor instrument with good psychometric properties (see for more information [132]. The tasks of the ZNA3–5 [67] include adaptive and pure motor tasks, static and dynamic balance tasks. Furthermore, during the performance of these tasks the associated movements are determined which occur when the children are performing the motor test. The tasks are demonstrated prior to the testing and explained verbally. For the time-based tasks, we measured how long the child takes for a certain movement, while for the dynamic motor tasks; performance is quantified on a 5-point scale. The tests will be recorded on video and analyzed off line. A total sum score is calculated for the quantification of fine and gross motor skill performance as well as for the associated movements which are a measure of neurological maturity of the child (see [67]).

Furthermore, environmental factors such as parenting style and family atmosphere is assessed through parents’ ratings. All parents are asked to complete the APQ [100, 133] to assess parenting style and will be interviewed to assess family atmosphere (FMSS) [104], especially expressed emotions in the family. During this interview parents are asked to describe general thoughts and attitudes towards the child in a free, unstructured manner. The standardized introduction is: “I would like you to speak for five minutes about your thoughts and feelings about (child’s name) in general without my interrupting”. The interview is audiotaped and parent’s expressed emotions (EE) in terms of emotional over-involvement and critical comments are coded.

Besides this, parents are asked about familial socioeconomic status (parental education, occupation, work load, income, place of birth), parental role modeling (e.g., sports club participation, being active with the children, parental BMI), health attitude and support (e.g., availability of PA equipment for a healthy lifestyle, lifestyle of the child including physical activity, sport club, screen time and sleep duration), chronic illness and any medical illness or accident which necessitate medical attention and/or hospitalization; passive smoking exposure and general home situation using the general health questionnaire. This questionnaire also includes questions about the pre-, peri- and postnatal conditions. These questions focus on pregnancy complications (e.g., premature contractions, vaginal bleeding, intrauterine growth retardation or other maternal medical problems, use of steroids or tocolytics, delivery problems), birth weight, gestational age, postpartum problems needing medical help, breastfeeding and early regulatory problems (e.g., crying, fussing etc.).

To clarify the role of child care settings, educators of each child care centers are asked to complete a questionnaire which focus on child care quality and health promotion. This includes the general child care quality [134], time spent in child care and health promoting activities (participating or not in Youp’là bouge/Purzelbaum). Further, information is collected about the parental home situation and the child’s social network within the child care center including behavior and playmates on child care days. Furthermore, sociocultural aspects including recruitment site (French or German speaking area) and child care center characteristics (rural or urban level and low-high SES levels of the child care center) are reported for all child care centers.

### Ethical considerations

The study protocol was approved by the Ethical Committee of the Canton of Vaud as the main applicant (No 338/13) and those of the other testing sites (Northwestern and Central of Switzerland, Berne, Fribourg and Zurich) and is in accordance with the Declaration of Helsinki. All parents receive written information about the aim of the study, benefits and risks of participation and the exact study procedure before giving their written informed consent to participate in the study. At that time all participants are informed that they can cancel participation without disclosing any reason at any time during the study.

Participation is not related to any physical or psychological risk. All assessments are well proven and currently used in the clinical settings and in other studies. The participating children receive small presents on each of the testing afternoons (including t-shirts, soft toy, ball, stickers, children tattoos etc.). Furthermore, all parents receive CHF 200 for each period of testings. The study has been registered at the ISRCTN registry (no. ISRCTN41045021, DOI 10.1186/ISRCTN41045021).

### Statistical analysis

The main analyses will be carried out according to the study analysis plan. First, descriptive statistics will be used for child care centers’ and children’s characteristics. Analyses will be adjusted for participants’ differences and/or cluster baseline characteristics i.e. age, gender and for further covariates such as SES depending on the respective research question. Multilevel models will be used to analyse the main outcomes of the study with child care center as upper and child as lower hierarchical level. For certain analyses, missing values are dealt with using either multiple imputation or maximum likelihood procedures which have repeatedly shown to provide unbiased parameter estimates under the missing at random (MAR) condition [135]. Internal validation will be used for assessing the self-developed Picture Stress Test. Further, longitudinal data will be analysed using multilevel models with child as upper and time within child as lower hierarchical level. This model can be extended by including child care center as an additional uppermost level if appropriate.

## Discussion

This study examines the influence of stress and physical activity on children’s psychological (cognitive functioning, psychological well-being) and physiological (adiposity and motor skills) health. So far, no longitudinal study exists that specifically evaluates the influence of these factors in children at preschool age. Given the fact that children’s health needs to be improved to diminish illness and risk factors, the detection of the influence of potentially challenging and health promoting predictors at different stages of early childhood is of high importance. Moreover, investigating the role of child’s and environmental characteristics and their effect on stress regulation might help to specify the mechanisms related to stress reactivity in early childhood. Additionally, the relationship between the child’s psychological and physiological health factors will be investigated and the potential sensitive periods where certain fundamental health indicators are particularly receptive (e.g., for physical activity promotion) or vulnerable (e.g., to stress exposure) can be identified according to the longitudinal assessment.

There are several strengths of the study. Firstly, a community-based longitudinal healthy cohort of children in Switzerland will be assessed by including children from important socio-culturally diverse regions starting at an early and sensitive stage of their childhood. Secondly, very relevant challenging and health promoting factors (stress and physical activity) on children’s health condition will be assessed and its influence on the child’s development will be investigated in all facets (i.e. motor skills, cognitive skills, emotional skills, social skills). Thirdly, the concept of stress used in this study integrates a time dimension (acute and chronic stressors) and a dual assessment approach (psychological and physiological stress response) which allows identifying a differentiated stress reaction. Forthly, most of the measurements used are objective and well-validated. By including relevant moderators which might explain the variance of physical activity and stress enables the identification of personal sensitivity and the impact of the context and further allows to promote the development of tailored interventions. Furthermore, the combination of a laboratory and observational design within this project and the direct measurements on one hand including novel and partly explorative parameters (i.e. salivary alpha- amylase, cortisol in fingernails and HRV in preschool children, assessment of nonverbal early correlates of emotion regulation) and on the other hand in combination with a multi-informant approach allows increased validity of the data.

There are also limitations of the study design. Assessment of physiological measures (i.e. salivary alpha- amylase, cortisol levels in nails and HRV monitoring) and assessment of subjective stress perception (PST) at this age is novel and cannot be compared with earlier studies, but will help to understand stress conditions in preschool children.

In conclusion, up to now, it remains open how relevant determinants of today’s life (stress and physical activity) in combination or interaction with different child’s and environmental characteristics, influence psychological and physiological health at different time points of childhood. A global assessment of children’s health at the early stage of childhood and longitudinal data collection during childhood is a basis for further evidence of vulnerable stages and therefore for targeted prevention and early treatment.

### Trial status

Recruitment of participants, beginning in March 2014, ended in December 2015, as new children were invited to participate in this second wave. Participant’s data collection is ongoing.

## Abbreviations

ANS, Autonomic nervous system; APQ, Alabama Parenting Questionnaire; BMI, Body mass index; CC, Child care center; CEBQ, Children’s Eating Behaviour Questionnaire; CNS, Central nervous system; EAS, Emotionality Activity Sociability Temperament Survey; ES, Effect size; FMSS, Parental expressed emotions Five Minute Speech Sample; HPA, Hypothalamic-pituitary-adrenal axis; HR, Heart rate; HRV, Heart rate variability; IDS-P, Intelligence and Development Scales- Preschool; NEPSY, Statue test NEPSY; PSS, Parenting Stress Scale; PST, Picture Stress Test; SDQ, Strengths and Difficulties Questionnaire; SES, Socioeconomic status; SNS, Sympathetic nervous system; ZNA3-5, Zurich Neuromotor Assessment
